# Comprehensive molecular characterization of TFE3-rearranged renal cell carcinoma

**DOI:** 10.1038/s12276-024-01291-2

**Published:** 2024-08-01

**Authors:** Cho-Rong Lee, Jungyo Suh, Dongjun Jang, Bo-Yeong Jin, Jaeso Cho, Moses Lee, Hyungtai Sim, Minyong Kang, Jueun Lee, Ju Hyun Park, Kyoung-Hwa Lee, Geum-Sook Hwang, Kyung Chul Moon, Cheryn Song, Ja Hyeon Ku, Cheol Kwak, Hyeon Hoe Kim, Sung-Yup Cho, Murim Choi, Chang Wook Jeong

**Affiliations:** 1 Department of Biomedical Sciences, Seoul National University College of Medicine, Seoul, Republic of Korea; 2 Department of Urology, Seoul National University Hospital, Seoul National University College of Medicine, Seoul, Republic of Korea; 3 Department of Urology, Asan Medical Center, University of Ulsan College of Medicine, Seoul, Republic of Korea; 4 Department of Biochemistry, Seoul National University College of Medicine, Seoul, Republic of Korea; 5 Department of Urology, Samsung Medical Center, Sungkyunkwan University School of Medicine, Seoul, Republic of Korea; 6 Samsung Genome Institute, Samsung Medical Center, Seoul, Korea; 7 Department of Health Sciences and Technology, SAIHST, Sungkyunkwan University, Seoul, Korea; 8 Department of Digital Health, SAIHST, Sungkyunkwan University, Seoul, Korea; 9 Integrated Metabolomics Research Group, Western Seoul Center, Korea Basic Science Institute, Seoul, Republic of Korea; 10 Department of Pharmacy, Chung-Ang University, Seoul, Republic of Korea; 11 Songdo Bio-Engineering, Incheon Jaeneung University, Incheon, Republic of Korea; 12 Department of Pathology, Seoul National University Hospital, Seoul National University College of Medicine, Seoul, Republic of Korea; 13 Medical Research Center, Genomic Medicine Institute, Seoul National University College of Medicine, Seoul, Republic of Korea; 14 Cancer Research Institute, Seoul National University, Seoul, Republic of Korea

**Keywords:** Translational research, Cancer genomics

## Abstract

*TFE3*-rearranged renal cell cancer (tRCC) is a rare form of RCC that involves chromosomal translocation of the Xp11.2 *TFE3* gene. Despite its early onset and poor prognosis, the molecular mechanisms of the pathogenesis of tRCC remain elusive. This study aimed to identify novel therapeutic targets for patients with primary and recurrent tRCC. We collected 19 *TFE3*-positive RCC tissues that were diagnosed by immunohistochemistry and subjected them to genetic characterization to examine their genomic and transcriptomic features. Tumor-specific signatures were extracted using whole exome sequencing (WES) and RNA sequencing (RNA-seq) data, and the functional consequences were analyzed in a cell line with *TFE3* translocation. Both a low burden of somatic single nucleotide variants (SNVs) and a positive correlation between the number of somatic variants and age of onset were observed. Transcriptome analysis revealed that four samples (21.1%) lacked the expected fusion event and clustered with the genomic profiles of clear cell RCC (ccRCC) tissues. The fusion event also demonstrated an enrichment of upregulated genes associated with mitochondrial respiration compared with ccRCC expression profiles. Comparison of the RNA expression profile with the *TFE3* ChIP-seq pattern data indicated that *PPARGC1A* is a metabolic regulator of the oncogenic process. Cell proliferation was reduced when *PPARGC1A* and its related metabolic pathways were repressed by its inhibitor SR-18292. In conclusion, we demonstrate that PPARGC1A-mediated mitochondrial respiration can be considered a potential therapeutic target in tRCC. This study identifies an uncharacterized genetic profile of an RCC subtype with unique clinical features and provides therapeutic options specific to tRCC.

## PPARGC1A: a promising target for mitochondrial respiration in tRCC

Understanding the unique traits of a rare kidney cancer type, TFE3-rearranged renal cell carcinoma, is important due to its poor response to usual treatments. This study explores the genetic and metabolic makeup of tRCC, comparing it with clear cell RCC and normal kidney cells. Using a mix of cell culture, whole exome sequencing, and various molecular analyses, the team conducted an experiment to reveal the unique genetic and metabolic profiles of tRCC. The researchers conclude that targeting the metabolic changes in tRCC, specifically through inhibiting PPARGC1A-mediated mitochondrial respiration, offers a new treatment approach. This approach marks a significant step in understanding and potentially treating tRCC. The implications of this study could lead to more effective treatments for patients with this challenging cancer type, emphasizing the importance of metabolic pathways in cancer therapy.

This summary was initially drafted using artificial intelligence, then revised and fact-checked by the author.

## Introduction

Transcription factor E3 (TFE3) gene-rearrangement renal cell carcinoma (tRCC) is a rare subtype of kidney cancer characterized by chromosomal rearrangements involving the XP11 locus^1^. This subtype, previously known as Xp11.2 translocation RCC, is the most common subtype of the *MiT* family of translocation RCCs^2,3^. Although tRCC is most commonly diagnosed in children and young adults^4,5^, its incidence among older adults is increasing, and it is estimated to account for approximately 4% of adult RCC cases^6–8^. In many cases, tRCC is diagnosed in an advanced stage at presentation and is usually associated with unfavorable outcomes^9,10^. However, its pathophysiology and genetic characteristics remain poorly understood^3^. Unfortunately, tRCC is not responsive to standard treatments, and no approved treatment options are available^10,11^.

Modifications and adaptations in cellular metabolism are hallmarks of cancer cells. After the pioneering discovery made by Otto Warburg showing that cancer cells preferentially use glycolysis to meet their energetic needs, several studies have shown that cancer cells adopt alternative metabolic methods for survival^12^. Recent findings have elucidated the metabolic changes, also termed metabolic reprogramming, that are observed in cancer cells in response to environmental challenges^13,14^. One major, well-described cell metabolism modulator is peroxisome proliferator-activated receptor gamma coactivator 1 (*PPARGC1A*), which regulates mitochondrial biogenesis and oxidative metabolism^15^.

To better understand the molecular landscape of tRCC and identify potential therapeutic targets, we performed a genetic analysis of tRCC patients diagnosed based on TFE3 overexpression using immunohistochemical (IHC) staining^16^. We observed distinct genetic and metabolic profiles between clear cell renal cell carcinoma (ccRCC) and normal kidney cells. Furthermore, the results of this study demonstrate that a metabolic mediator of TFE3 activation is involved in tRCC.

## Materials and methods

### Cell culture and transfection

The human renal clear cell carcinoma cell line Caki-1 and the Xp11.2 translocation renal cell carcinoma cell lines (UOK109, UOK120, UOK124, UOK145, and UOK146) were maintained in DMEM (Cytiva, Marlborough, MA). The human renal proximal tubular epithelial cell line HK-2 was maintained in DMEM/F-12 medium (Biowest, Bradenton, FL). All media were supplemented with 10% FBS (Cytiva, Marlborough, MA) and 1% penicillin‒streptomycin (Thermo Fisher Scientific, Waltham, MA). All cells were incubated at 37 °C in a humidified atmosphere containing 5% CO_2_. Transfection was performed with short interfering RNAs (siRNAs) against *TFE3* and *PPARGC1A* (Bioneer, Daejeon, Korea) using Lipofectamine RNAiMAX transfection reagents (Thermo Fisher, Waltham, MA) following the manufacturer’s instructions.

### Whole exome sequencing (WES) and acquisition and processing of RNA-seq data

DNA and RNA samples were prepared from the tumor and blood tissues of participants by standard procedures and were processed at the Theragen Etex Bio Institute (Suwon, Korea) for WES. The reads were processed according to GATK 4.0 Best Practices for somatic single nucleotide variant (SNV) and structural change analysis^17^. RNA-seq reads were aligned by STAR aligner, and DESeq2 was used to identify differentially expressed genes (DEGs)^18^. The data related to the gene fusion events for TCGA samples were obtained from the TCGA Fusion Gene Database^19^. The MR4Cancer tool was used to identify nontranscriptional master regulators in our dataset based on the TCGA kidney renal clear cell carcinoma (KIRC) dataset^20^. To identify a possible master regulator of the input DEG set, each set was divided into two subsets based on either a positive or a negative Spearman correlation of the expression of the regulon genes.

### Chromatin immunoprecipitation (ChIP)-seq

UOK146 cells (tRCC with a *PRCC-TFE3* fusion) were fixed with 1% formaldehyde and subjected to ChIP as previously described^21^ using an antibody against TFE3 (Sigma‒Aldrich, HPA023881). ChIP-seq data were mapped to the human genome (hg19) using the Bowtie algorithm, allowing up to two mismatches. Reads mapped to more than 20 locations along the genome were discarded. ChIP-seq data generated using an IgG antibody were used as a control^22^. Peaks were identified using MACS2 with an FDR-adjusted *P* value cutoff of 0.05. Two biological replicates were generated^23^.

### Chromatin immunoprecipitation (ChIP)-qPCR

ChIP assays were performed using the SimpleChIP Enzymatic Chromatin IP Kit (Cell Signaling Technology, Danvers, MA) according to the manufacturer’s instructions. UOK146 cells were cross-linked with 1% formaldehyde for 10 min, and chromatin was extracted using 1 M DTT. The prepared chromatin was digested with micrococcal nuclease and sonicated to obtain fragments of approximately 150–900 base pairs. Immunoprecipitation was conducted with either an antibody against TFE3 (Abcam, ab93808) or IgG using protein G magnetic beads at 4 °C. Following immunoprecipitation, the beads were washed and reversely cross-linked, and the eluted DNA was purified. Purified DNA samples, along with input DNA samples, were subjected to quantitative real-time PCR (qRT‒PCR) analysis using SYBR^®^ Green Master Mix (Bio‒Rad, Hercules, CA). Primers specific to the *PPARGC1A* promoter were designed to amplify the regions corresponding to two distinct peaks identified in the ChIP-seq data. The primer sequences for peak 1 were 5′-GGGAAGGTTAAGTGGGTGGT-3′ (forward) and 5′-TCCTGCATAGCACAGTGGAG-3′ (reverse), and those for peak 2 were 5′-GGTTCTGCCTGGAGTTGTTC-3′ (forward) and 5′-CCATCGCTAGCTTTCCAGTC-3′ (reverse).

### Mitochondrial mass and cell proliferation

siRNAs against *TFE3* and *PPARGC1A* (Bioneer, Daejeon, Korea) were transfected into UOK146 cells using Lipofectamine RNAiMAX transfection reagents (Thermo Fisher, Waltham, MA) in 6-well plates at concentrations ranging from 10–80 nM. The knockdown efficiency was checked after 72 h. At 24 h posttransfection, the cells were plated in 96-well plates at a density of 1 × 10^4^ cells/well and incubated in the presence or absence of SR18292 (60 µM) or oligomycin (20 µM). After 48 h, cell proliferation was evaluated with a Cell Counting Kit-8 (CCK8; Dojindo, Japan) according to the manufacturer’s instructions. The absorbance was measured at 450 nm using a Glomax Discover System (Promega, Madison, WI).

Mitochondrial mass was analyzed by flow cytometry in cells labeled with MitoTracker Deep Red FM (M22426, Thermo Fisher Scientific, Waltham, MA). Forty-eight hours after transfection, the cells were incubated in warm PBS containing 50 nM MitoTracker Deep Red FM for 20 min at 37 °C. The samples were washed in PBS and then fixed with PBS containing 2.5% formaldehyde. Fluorescence was measured by a BD LSRII (BD Biosciences, San Jose, CA) flow cytometer. BD FACSDiva (BD Biosciences, San Jose, CA) software was used for data analysis.

### RNA extraction and qRT‒PCR

Total RNA was isolated from cells using the RNeasy Plus Mini Kit (Qiagen, Germany) according to the manufacturer’s instructions. Complementary DNA (cDNA) was synthesized from 0.5 μg of RNA using the PrimeScript RT reagent Kit (Takara Bio, Japan). qRT‒PCR was performed using SYBR^®^ Green Master Mix (Bio-Rad, Hercules, CA) on a Bio-Rad CFX96 instrument (Bio-Rad, Hercules, CA). Glyceraldehyde-3-phosphate dehydrogenase (GAPDH) was used as the endogenous control for mRNA normalization. Primer sequences used in qRT-PCR are listed in Supplementary Table 1.

### Protein extraction and western blotting

Cells were harvested and lysed using RIPA buffer (Thermo Fisher Scientific, Waltham, MA) supplemented with a protease inhibitor cocktail (Roche, Switzerland). After centrifugation, the protein concentration was determined using a BCA assay kit (Thermo Fisher Scientific, Waltham, MA) and mixed with sample buffer at 100 °C for 5 min. An equal amount of protein was separated via SDS‒PAGE and transferred onto NC membranes (Cytiva, Marlborough, MA). The membranes were blocked with 5% skim milk (BD Biosciences, San Jose, CA) in TBS-T buffer and subsequently incubated with primary and secondary antibodies. Protein bands were visualized using the SuperSignal™ West Pico PLUS ECL kit (Thermo Fisher Scientific, Waltham, MA) on an Amersham Imager AI680 (General Electric, Boston, MA). The following antibodies were used: anti-E-cadherin (Cell Signaling Technology, #3195), anti-N-cadherin (Cell Signaling Technology, #13116), anti-vimentin (Cell Signaling Technology, #5741), anti-α-SMA (Abcam, ab5694) and anti-β-actin (Sigma‒Aldrich, A5441).

### Migration assay

Cells in serum-free DMEM were reseeded into the upper chamber of a transwell insert (Corning, Corning, NY), and the lower chamber was filled with DMEM containing 10% FBS. After 24 h of incubation, the migrated cells in the upper chamber were fixed with cold methanol and stained with 1% crystal violet. The migrated cells were visualized and imaged using an EVOS XL Core microscope (Thermo Fisher Scientific, Waltham, MA).

## Results

We initially collected 19 RCC samples with TFE overexpression, as determined by IHC, six ccRCC tissue samples, and four normal kidney tissue samples. The median age of the 19 patients with TFE3-positive RCC was 45 years (interquartile range [IQR]: 40–52), and 11 patients (57.9%) were female (Table 1). Six patients (31.6%) had locally advanced disease, and four (21.1%) had distant metastasis. Seven patients (36.8%) underwent partial nephrectomy, and five patients (26.3%) underwent surgery via a laparoscopic approach. The mean size of the tumors was 5.0 cm (IQR: 3.3–6.7). Recurrence was observed in four patients, and the median recurrence-free survival was 31 months (IQR: 24–54).

**Table 1:** Clinical characteristics of 19 TFE3-positive RCCs.

	Value
Median age at diagnosis, year (IQR)	45 (40–52)
Female, *n* (%)	11 (57.9)
Diabetes, *n* (%)	1 (5.3)
Hypertension, *n* (%)	3 (15.8)
BMI, kg/m^2^ (IQR)	21.6 (20.7–23.2)
*Tumor location, n (%)*	
Right	11 (57.9)
Left	8 (42.1)
*Pathological T stage, n (%)*	
T 1/2	11 (57.9)
T 3/4	8 (42.1)
Node positive, *n* (%)	5 (26.3)
Distant metastasis, *n* (%)	4 (21.1)
*Operation methods, n (%)*	
Radical nephrectomy	12 (63.2)
Partial nephrectomy	7 (36.8)
Laparoscopic approach, *n* (%)	5 (26.3)
Tumor size, cm (IQR)	5.0 (3.3–6.7)
*Fuhrman nuclear grade, n (%)*	
1/2	1 (5.3)
3	14 (73.7)
4	4 (21.1)
Presence of lympho-vascular invasion, *n* (%)	5 (26.3)
Positive surgical margin, *n* (%)	2 (10.5)
Recurrence, *n* (%)	4 (21.1)
Recurrence free survival, mo (IQR)	31 (24–54)
Deceased, *n* (%)	3 (15.8)

*IQR* interquartile range, *mo* months.

**Fig. 1:**
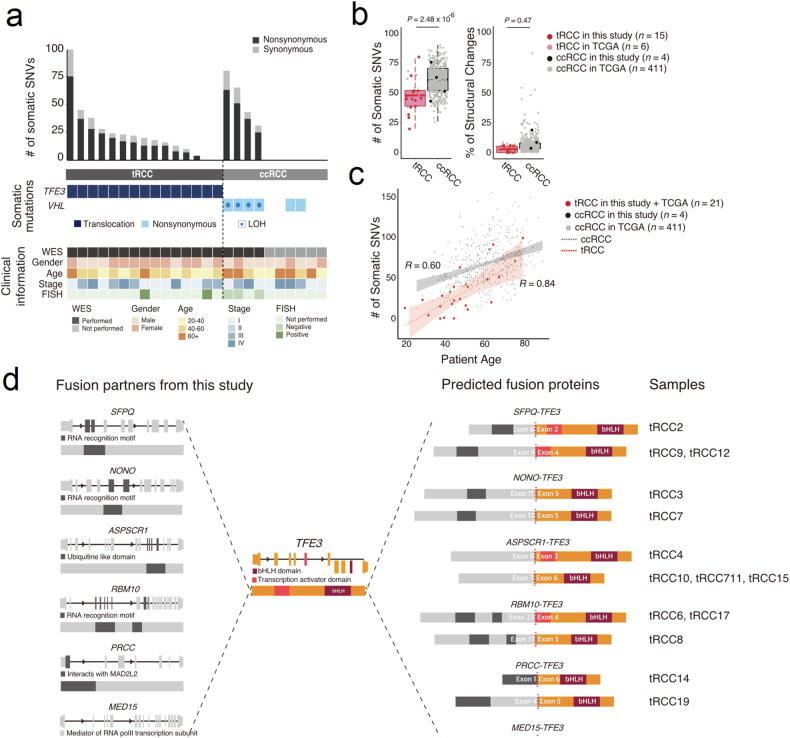
Genomic profile of tRCC. **a** A clinical and genomic overview of 15 pathologically confirmed tRCC and four ccRCC tumors. **b** Number of somatic SNVs and the genomic portion of structural changes between tRCC and ccRCC samples. **c** Differences in the correlation between the number of somatic SNVs and patient age. **d** Schematic diagram representing *TFE3* fusion events identified by RNA-seq. For each gene, the upper diagrams denote the gene structure with alternating exons and introns. The lower diagrams show the protein structure. tRCC translocation renal cell carcinoma, ccRCC clear cell renal cell carcinoma, SNVs single nucleotide variations, WES whole exome sequencing, FISH fluorescence in situ hybridization, TCGA The Cancer Genome Atlas, LOH loss of heterozygosity.

Among the 19 *TFE3*-positive specimens, four samples did not demonstrate a *TFE3* fusion according to the results of the confirmative fluorescence in situ hybridization (FISH) assay or RNA-seq analysis. Therefore, 15 samples with confirmed tRCC remained (Fig. 1a). tRCC tumors are sometimes misdiagnosed as ccRCC^24,25^. Therefore, we searched for such samples in the TCGA KIRC database by screening for *TFE3* translocation events via WES/RNA-seq. Among the 417 ccRCC samples, six harbored *TFE3* translocations^19^. There were fewer somatic mutations in tRCC than in ccRCC (45.6 vs. 58.3, *P* = 2.5 × 10^−6^, Student’s *t* test; Fig. 1b). *VHL* was identified in our four ccRCC samples (Fig. 1a) but not in any of the tRCC samples. The percentage of the genome affected by structural changes in the tRCC and ccRCC samples was comparable (5.7% vs. 7.8%, *P* = 0.47, Student’s *t* test; Fig. 1b). These observations indicate the presence of a strong oncogenic driver in tRCC. No variants or genes other than *TFE3* translocations have been observed recurrently, supporting this interpretation. In addition, compared to ccRCC (*R* = 0.60), tRCC was more strongly correlated (*R* = 0.84) with the number of somatic variants and the age of onset (Fig. 1c).

**Fig. 2:**
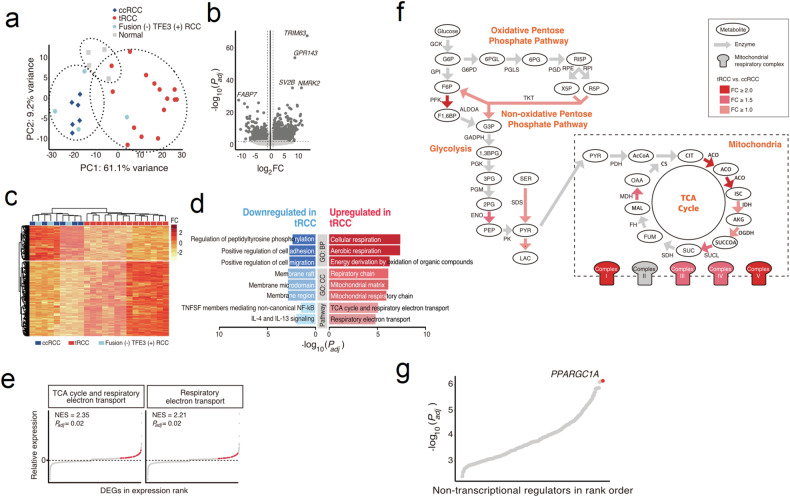
Transcriptomic profile of tRCC. **a** PCA plot of RCC and normal tissues. **b** Volcano plot, **c** Heatmap, **d** GO profiles, and **e** GSEA results obtained using DEGs in tRCC and ccRCC samples. **f** Pathways enriched with genes upregulated in tRCC. **g** Analysis of the master regulators of the DEGs. PCA principal component analysis, tRCC translocation renal cell carcinoma, ccRCC clear cell renal cell carcinoma, GO Gene Ontology, GSEA gene set enrichment analysis, DEGs differentially expressed genes, NES normalized enrichment score, TNFSF tumor necrosis factor superfamily, IL interleukin, TCA tricarboxylic acid, FC fold change.

Next, we performed RNA-seq analysis using fresh-frozen samples (19 tRCC, six ccRCC, and four normal kidney tissue samples). Based on the RNA-seq data, we identified six fusion partner genes of *TFE3* in 15 *TFE3* translocation-positive tRCC samples (Fig. 1d, Table 2, Supplementary Fig. 1). Principal component analysis (PCA) grouped the samples into three main clusters, which corresponded to tRCC, ccRCC, and normal tissues (Fig. 2a). Remarkably, the four tumors that were initially suspected to be tRCC but lacked *TFE3* fusion clustered with ccRCC, confirming that *TFE3* fusion is a strong determinant of tRCC. Comparison of the tRCC and ccRCC gene expression profiles revealed 2573 differentially expressed genes (DEGs) (Fig. 2b, c). Gene Ontology (GO) analysis of upregulated DEGs in tRCC compared to those in ccRCC revealed enrichment associated with metabolic pathways, such as the tricarboxylic acid (TCA) cycle, respiratory electron transport, and mitochondrial metabolic pathways. Genes with downregulated expression in tRCC included those related to cell adhesion and cell migration (Fig. 2d). Notably, the adaptive immune response pathway was downregulated, suggesting a diminished potential for a robust response to immunotherapy interventions. Gene set enrichment analysis (GSEA) of DEGs consistently revealed enrichment associated with metabolic pathways related to the TCA cycle and respiratory electron transport (Fig. 2e). These results indicate the presence of an oncogenic metabolic regulation pathway specific to tRCC. A closer look at the upregulated pathways revealed a unique metabolic environment that may lead to poor responses to conventional chemotherapies (Fig. 2f).

**Table 2:** Genetic and clinical features of the tumor samples used in this study.

Patient ID	Sex	Onset age	Tumor stage^a^	Histology	Driver mutation identified by WES	Fusion identified by RNA-seq	*TFE3* FISH results
tRCC 2	F	44	T3aN0M0	TFE3	–	*SFPQ-TFE3*	–
tRCC 3	M	52	T3aN0M0	TFE3	–	*NONO-TFE3*	–
tRCC 4	M	39	T1bN0M0	TFE3	–	*ASPSCR1-TFE3*	–
tRCC 6	M	37	T3aN0M0	TFE3	–	*RBM10-TFE3*	Fusion (+)
tRCC 7	F	51	T1aN0M0	TFE3	–	*NONO-TFE3*	–
tRCC 8	F	80	T1aN0M0	TFE3	–	*RBM10-TFE3*	–
tRCC 9	F	44	T1aN0M0	TFE3	–	*SFPQ-TFE3*	–
tRCC 10	F	47	T3aN0M0	TFE3	*SETD2* p.Gln1764Leu^c^	*ASPSCR1-TFE3*	–
tRCC 11	F	28	T3aN1M1	TFE3	–	*ASPSCR1-TFE3*	–
tRCC 12	M	45	T3aN1M1	TFE3	–	*SFPQ-TFE3*	–
tRCC 14	F	32	T1aN0M0	TFE3	–	*PRCC-TFE3*	–
tRCC 15	M	20	T1bN1M1	TFE3	–	*ASPSCR1-TFE3*	–
tRCC 17	F	45	T2N0M0	TFE3	–	*RBM10-TFE3*	–
tRCC 18	F	51	T1bN0M0	TFE3	–	*MED15-TFE3*	Fusion (+)
tRCC 19	F	64	T1bN0M0	TFE3	–	*PRCC-TFE3*	–
tRCC 1	M	62	T1bN0M0	TFE3	*PBRM1* p.Ser1057X^b^, *SETD2* p.Val2320Leu	TFE family*-*involved fusion not found	Fusion (-)
tRCC 5	F	75	T1bN1M0	TFE3	*VHL* p.His115Asn^b^		Fusion (−)
tRCC 13	M	51	T3aN0M1	TFE3	–		–
tRCC 16	M	43	T1bN0M0	TFE3	*VHL* p.Leu89His^b^		Fusion (−)
ccRCC 1	M	36	T1aN0M0	ccRCC	*VHL* p.S80-81del	TFE family*-*involved fusion not found	–
ccRCC 2	M	79	T4N0M1	ccRCC	*VHL* p.Leu89His^b^		–
ccRCC 3	F	71	T3aN0M1	ccRCC	–		–
ccRCC 4	M	61	T3aN0M0	ccRCC	–		–
ccRCC 5	M	78	T1aN0M0	ccRCC	–		–
ccRCC 6	F	37	T1bN0M0	ccRCC	–		–

^a^According to the AJCC TNM staging system.

^b^Also reported in COSMIC ccRCC samples.

^c^Also reported in other COSMIC cancer samples.

*tRCC* translocation renal cell carcinoma, *ccRCC* clear cell renal cell carcinoma.

To identify a possible downstream regulator of aberrant TFE3, a master regulator analysis was performed using our tRCC RNA-seq results with ARACNe^26^. The output was then compared to an output generated using ccRCC data from the TCGA KIRC cohort. The analysis revealed *PPARGC1A* as the top nontranscriptional regulator in the tRCC RNA-seq data (Fig. 2g). *PPARGC1A* encodes a transcriptional coactivator for steroid and nuclear receptors that plays an essential role in metabolic reprogramming^15^. This result suggests that *PPARGC1A* may play an integral role in the metabolic reprogramming of tRCC oncogenic processes.

**Fig. 3:**
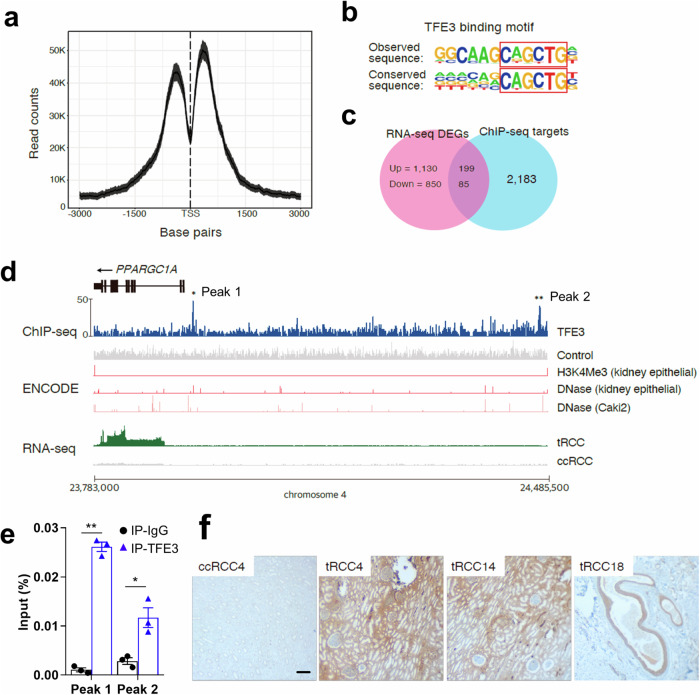
Identification of *PPARGC1A* as a regulator of tRCC. **a** Profile of TFE3 ChIP-seq signals relative to the TSS determined using the UOK146 cell line. **b** Motif analysis of the TFE3 ChIP-seq results. **c** Comparison of RNA-seq and ChIP-seq analyses. **d** TFE3 binding sites in the *PPARGC1A* upstream region. *P = 1.3 × 10^−^^9^. **P = 8.1 × 10^−14^. **e** ChIP‒qPCR analysis of the TFE3 binding sites upstream of *PPARGC1A*. ChIP‒qPCR was applied to amplify chromatin immunoprecipitated from the *PPARGC1A* gene promoter with an anti-TFE3 antibody using two independent sets of primers for peaks 1 and 2 in (**d**). *, 0.001 < *P* < 0.05. **, *P* < 0.001. **f** Immunohistochemical analysis of PPARGC1A in tumor tissues used for genome analysis. Scale bar = 200 µm. ChIP chromatin immunoprecipitation, TSS transcription start site, DEG differentially expressed gene, tRCC translocation renal cell carcinoma, ccRCC clear cell renal cell carcinoma.

To identify the genomic targets of the fused TFE3 gene, TFE3 ChIP-seq was performed using the UOK146 cell line, which harbors a *PRCC*-*TFE3* gene fusion^27^. Two biological replicates resulted in 6952 overlapping peaks enriched around transcription start sites (TSSs; Fig. 3a). Motif analysis revealed strong enrichment in CAGCTG sequences (*P* < 1.0 × 10^−24^), which completely overlapped with the known TFE3 binding motif (Fig. 3b)^28^. A comparison of DEGs from the RNA-seq experiment and TFE3 ChIP-seq data yielded 284 genes, including *PPARGC1A* (Fig. 3c). To evaluate whether the TFE3 fusion gene is associated with the regulation of *PPARGC1A*, we identified two TFE3 binding elements near the TSS of *PPARGC1A* (Fig. 3d, e and Supplementary Table 2). Combined with the master regulator analysis results (Fig. 2g), we observed that unique metabolic processes of tRCC could be regulated by the fusion of *TFE3*, resulting in the upregulation of *PPARGC1A* expression. To confirm whether the expression of PPARGC1A was indeed increased in *TFE3* translocation-positive tRCC, the tumor tissues were stained with a PPARGC1A-specific antibody. The majority of tRCC samples showed increased expression of PPARGC1A (0/3 normal, 0/3 ccRCC, and 7/8 in tRCC samples; Fig. 3f). In addition, reanalysis of RNA-seq data from a previous study revealed upregulated *PPARGC1A* in cells with *TFE3* fusion (Supplementary Fig. 2)^29^.

**Fig. 4:**
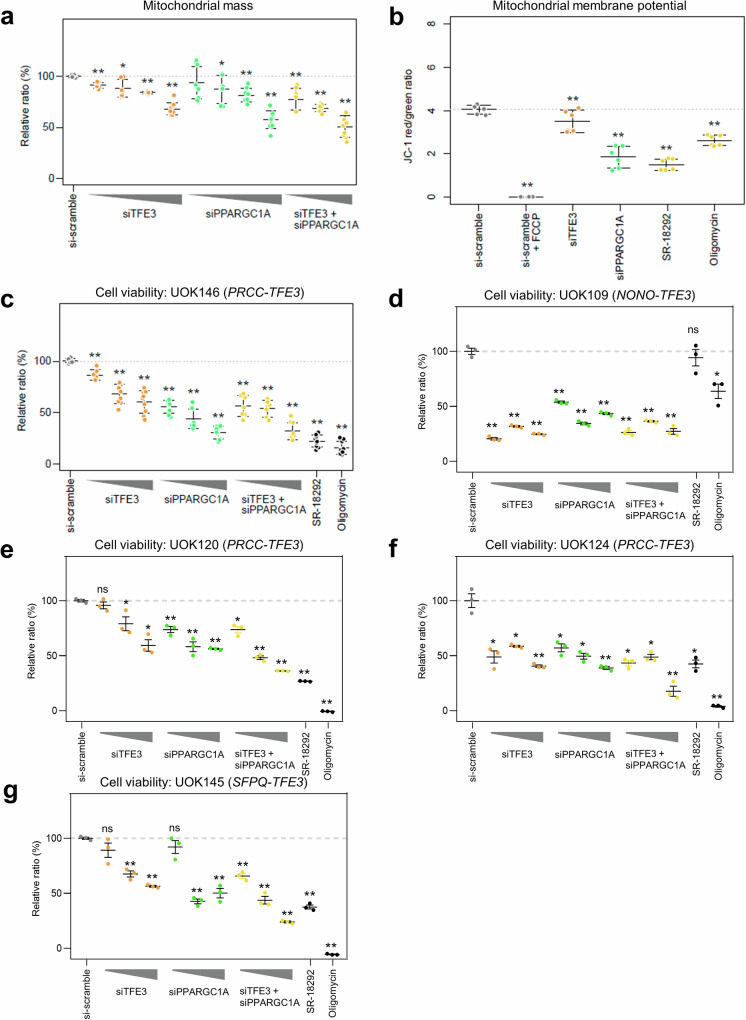
tRCC cell viability is reduced upon mitochondrial inhibition. **a** Relative mitochondrial mass after knocking down *TFE3*, *PPARGC1A*, or both in UOK146 cells. **b** Relative ratio of red versus green JC-1 signals. FCCP was used as a positive control in UOK146 cells. **c**–**g** Relative cell viability after downregulation of *TFE3*, *PPARGC1A*, or both in UOK146 (**c**), UOK109 (**d**), UOK120 (**e**), UOK124 (**f**), and UOK145 (**g**) cells. Inhibitors targeting mitochondrial function (oligomycin) and PPARGC1A (SR-18292) were also applied. *0.001 < *P* < 0.05. **P < 0.001.

These findings demonstrate that PPARCG1A is a strong mediator of TFE3-mediated increases in the TCA cycle and related pathways. Thus, we tested whether treatment of cells with reduced expression of *TFE3*, *PPARGC1A*, or mitochondrial inhibitors impacted their survival. Downregulation of *TFE3*, *PPARGC1A*, or both significantly reduced mitochondrial mass and membrane potential (Fig. 4a, b). A more potent effect on cell viability was observed upon treatment with a selective *PPARGC1A* inhibitor, SR-18292^30^ and the mitochondrial respiration inhibitor oligomycin in tRCC cell lines with various *TFE3* fusions (Fig. 4c–g). This effect was not observed in the normal kidney cell line. Although the effect on ccRCC cells may be dependent on *VHL* mutation status (Supplementary Fig. 3), these results indicate that *PPARGC1A* is a key regulator of mitochondrial respiration and cell proliferation in tRCC.

**Fig. 5:**
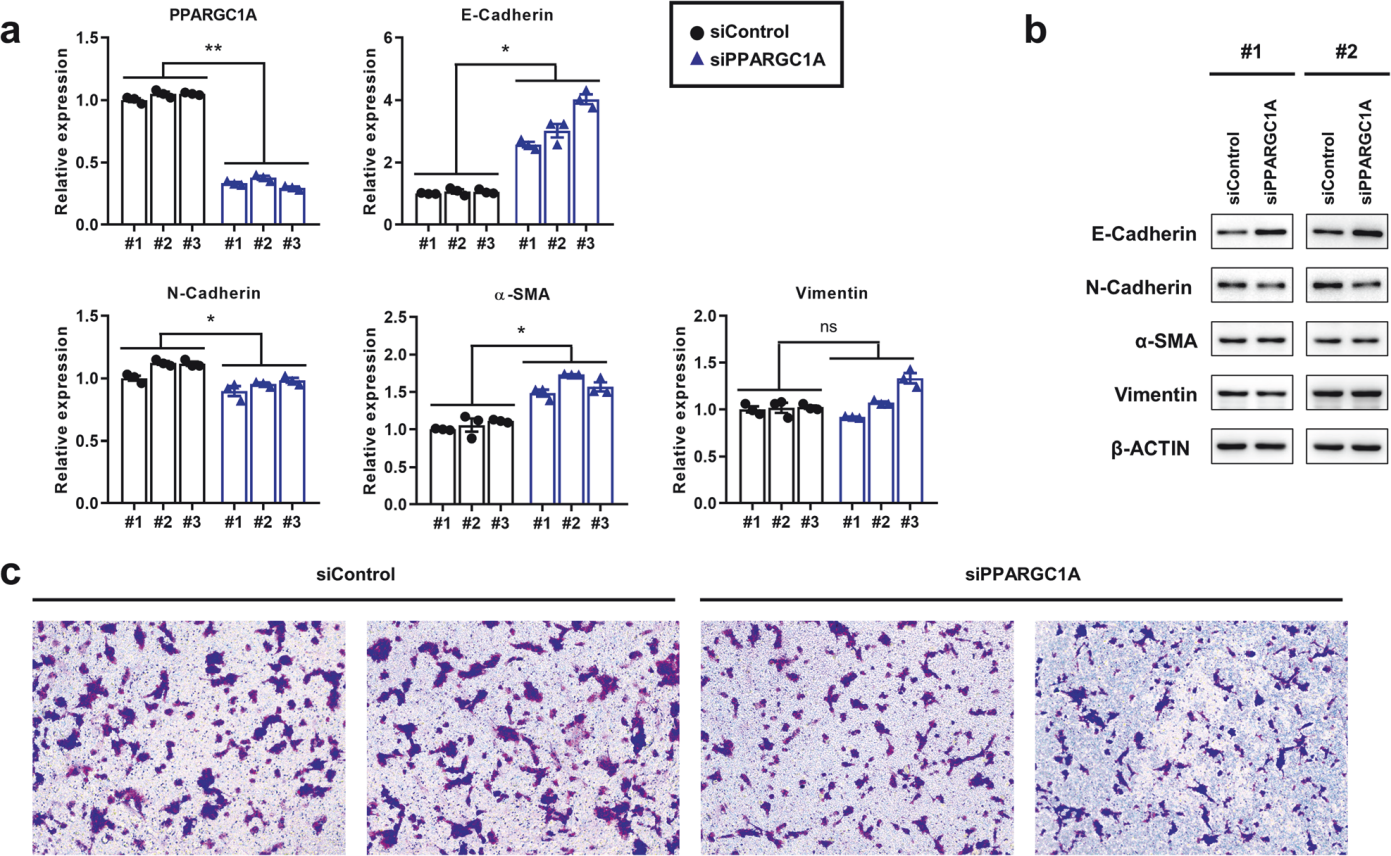
Depletion of *PPARGC1A* reduces EMT in tRCC cells. **a**, **b** EMT markers after knocking down *PPARGC1A* at the mRNA (**a**) and protein (**b**) levels. The mRNA and protein expression levels of EMT markers were evaluated by real-time PCR and western blotting, respectively. *0.001 < *P* < 0.05. **P < 0.001. **c** Cell migration analysis after knocking down *PPARGC1A* in UOK146 cells. In vitro cell migration was assessed using transfilter migration assays; representative images of migrated cells are shown. siControl control siRNA, siPPARGC1A siRNA for *PPARGC1A*.

Finally, we investigated how the TFE3-mediated modulation of metabolic pathways affects the cancer phenotype. We altered *PPARGC1A* expression and investigated its effects on cancer aggressiveness. Treatment of UOK146 cells with siPPARGC1A upregulated E-cadherin, an epithelial marker, and downregulated N-cadherin, a mesenchymal marker, at both the mRNA and protein levels (Fig. 5a, b). Additionally, knockdown of PPARGC1A reduced the migration of UOK146 cells (Fig. 5c). These findings suggest that the TFE3-mediated modulation of the PPARGC1A-mediated modulation of metabolic pathways plays a role in epithelial–mesenchymal transition (EMT) and cancer aggressiveness.

## Discussion

It is estimated that tRCC comprises approximately one-third of pediatric RCC cases and 15% of RCC cases in patients aged 45 years or younger^4,7^. Our group previously identified 61 patients out of 8384 consecutive patients with RCC (0.7%) in the largest multicenter study to date^31^. Despite the increased recognition of tRCC, its diagnosis remains challenging. tRCCs typically display papillary and/or alveolar architecture and are composed of cells with voluminous eosinophilic and/or clear cytoplasm^2,6,32^. Therefore, they can be histologically confused with papillary or ccRCCs. Indeed, *TFE3* fusions were identified in six ccRCC samples and ten papillary RCC samples out of 417 KIRC and 289 KIRP TCGA samples, respectively^19^. This finding indicates that tRCCs are frequently misclassified as ccRCC or papillary RCC without accurately evaluating fusion events. The overexpression of *TFE3* can be detected by IHC^32^, which is widely used as a diagnostic tool. However, accumulating evidence suggests that IHC-based diagnosis of tRCC may generate false-positives^33^. Our study had a 21.1% (4/19) false-positive rate in immunohistochemical diagnosis, as these falsely diagnosed patients showed greater genetic resemblance to patients with ccRCC than to those with tRCC. This result was confirmed by the presence of *VHL* mutations (Fig. 1a) and PCA of the RNA-seq data (Fig. 2a). These patients were also older than patients with *TFE3* fusions, and their oncological outcomes were inferior to those of the other patients. Our comprehensive genomic profiling of tRCC tumor tissues revealed fewer somatic mutations in tRCC than in ccRCC, suggesting that tRCC is strongly associated with oncogenes (Fig. 1b). Our transcriptomic profiling of tRCC tissues revealed that tRCC is associated with a unique metabolic profile that is highly enriched in mitochondrial respiration and the TCA cycle. Our transcriptomic analysis identified *PPARGC1A* as a master regulator of the transcriptomic changes observed in tRCC tissues (Fig. 2g). Subsequent ChIP-seq analysis using a tRCC cell line (UOK146) demonstrated that TFE3 binds to the *PPARGC1A* promoter to upregulate DEGs more often than to downregulate DEGs (Fig. 3d, e). These findings indicate a gain-of-function effect of the *TFE3* fusion, resulting in unique mitochondria-focused metabolic reprogramming via activation of *PPARGC1A*. In addition, *TFE3*-overexpressing cells and cells harboring various *TFE3* fusions clustered together, supporting our conclusion^29^.

Many glycolytic tumor cells rely on lactate production to generate NAD+ for their anabolic metabolism (known as the Warburg effect)^34^. Our analyses suggest that tRCC tumors are highly dependent on mitochondrial respiration for energy. The dependence on oxidative phosphorylation for energy production is consistent with that of leukemic stem cells^35^ and surviving pancreatic cancer cells^12^. The inability of cells to compensate for mitochondrial energy deprivation by increasing glycolysis renders them highly vulnerable to various therapeutics that inhibit oxidative phosphorylation. Although *MTOR* inhibitors have been identified as potential targets for treating tRCC^36,37^, this signal appeared only when we compared matched tumor and normal samples (BP:0002224, *P*_*adj*_ = 1.5 × 10^−5^), suggesting that the MTOR signaling pathway is less prominent than the metabolic pathway that we investigated (Supplementary Figs. 4 and 5).

Through the most comprehensive integrated genetic study to date using 19 *TFE3*-positive RCC cases, we provide a genomic landscape and a deeper understanding of the oncogenic mechanism of tRCC, facilitating further discovery of a therapeutic strategy for tRCC. Our results indicate that inhibitors of mitochondrial function or PPARGC1A could be efficiently utilized as monotherapy or combination therapy for treating tRCC.

In conclusion, we have demonstrated that PPARGC1A-mediated mitochondrial respiration can be considered a potential therapeutic target in tRCC. This study has identified an uncharacterized genetic profile of an RCC subtype with unique clinical features and provides therapeutic options specific to tRCC.

## Supplementary information

Supplementary materials

## Data Availability

The data generated in this study are available upon request from the corresponding author.

## References

[1] Wei, S. et al. Molecular characterization of TFE3-rearranged renal cell carcinoma: a comparative study with papillary and clear cell renal cell carcinomas. *Mod. Pathol.* **37**, 100404 (2024). 38104891 10.1016/j.modpat.2023.100404

[2] Argani, P. MiT family translocation renal cell carcinoma. *Semin. Diagn. Pathol.* **32**, 103–113 (2015). 25758327 10.1053/j.semdp.2015.02.003

[3] Kauffman, E. C. et al. Molecular genetics and cellular features of TFE3 and TFEB fusion kidney cancers. *Nat. Rev. Urol.* **11**, 465–475 (2014). 25048860 10.1038/nrurol.2014.162 PMC4551450

[4] Malouf, G. G. et al. Transcription factor E3 and transcription factor EB renal cell carcinomas: clinical features, biological behavior and prognostic factors. *J. Urol.* **185**, 24–29 (2011). 21074195 10.1016/j.juro.2010.08.092

[5] Qiu Rao et al. Renal cell carcinoma in children and young adults: clinicopathological, immunohistochemical, and VHL gene analysis of 46 cases with follow-up. *Int. J. Surg. Pathol.* **19**, 170–179 (2011). 20034980 10.1177/1066896909354337

[6] Argani, P. et al. Xp11 translocation renal cell carcinoma in adults: expanded clinical, pathologic, and genetic spectrum. *Am. J. Surg. Pathol.* **31**, 1149–1160 (2007). 17667536 10.1097/PAS.0b013e318031ffff

[7] Komai, Y. et al. Adult Xp11 translocation renal cell carcinoma diagnosed by cytogenetics and immunohistochemistry. *Clin. Cancer Res.* **15**, 1170–1176 (2009). 19228722 10.1158/1078-0432.CCR-08-1183

[8] Zhong, M. et al. Dual-color, break-apart FISH assay on paraffin-embedded tissues as an adjunct to diagnosis of Xp11 translocation renal cell carcinoma and alveolar soft part sarcoma. *Am. J. Surg. Pathol.* **34**, 757–766 (2010). 20421778 10.1097/PAS.0b013e3181dd577e PMC7386799

[9] Boilève, A. et al. Immune checkpoint inhibitors in MITF family translocation renal cell carcinomas and genetic correlates of exceptional responders. *J. Immunother. Cancer* **6**, 159 (2018). 30591082 10.1186/s40425-018-0482-z PMC6307255

[10] Kakoki, K. et al. Long-term treatment with sequential molecular targeted therapy for Xp11.2 translocation renal cell carcinoma: a case report and review of the literature. *Clin. Genitourin. Cancer* **15**, e503–e506 (2017). 28190703 10.1016/j.clgc.2016.12.026

[11] Choueiri, T. K. et al. Vascular endothelial growth factor-targeted therapy for the treatment of adult metastatic Xp11.2 translocation renal cell carcinoma. *Cancer* **116**, 5219–5225 (2010). 20665500 10.1002/cncr.25512 PMC4667556

[12] Viale, A. et al. Oncogene ablation-resistant pancreatic cancer cells depend on mitochondrial function. *Nature* **514**, 628–632 (2014). 25119024 10.1038/nature13611 PMC4376130

[13] Lee, H. & Yoon, H. Mitochondrial sirtuins: energy dynamics and cancer metabolism. *Mol. Cells* **47**, 100029 (2024). 38331199 10.1016/j.mocell.2024.100029 PMC10960136

[14] Martínez-Reyes, I. & Chandel, N. S. Cancer metabolism: looking forward. *Nat. Rev. Cancer* **21**, 669–680 (2021). 34272515 10.1038/s41568-021-00378-6

[15] Schreiber, S. N., Knutti, D., Brogli, K., Uhlmann, T. & Kralli, A. The transcriptional coactivator PGC-1 regulates the expression and activity of the orphan nuclear receptor estrogen-related receptor α (ERRα). *J. Biol. Chem.* **278**, 9013–9018 (2003). 12522104 10.1074/jbc.M212923200

[16] Akgul, M. et al. Diagnostic approach in TFE3-rearranged renal cell carcinoma: a multi-institutional international survey. *J. Clin. Pathol.* **74**, 291–299 (2021). 33514585 10.1136/jclinpath-2020-207372

[17] Poplin, R. et al. *Scaling Accurate Genetic Variant Discovery to Tens of Thousands of Samples*. http://biorxiv.org/lookup/doi/10.1101/201178 (2017).

[18] Love, M. I., Huber, W. & Anders, S. Moderated estimation of fold change and dispersion for RNA-seq data with DESeq2. *Genome Biol.* **15**, 550 (2014). 25516281 10.1186/s13059-014-0550-8 PMC4302049

[19] Hu, X. et al. TumorFusions: an integrative resource for cancer-associated transcript fusions. *Nucleic Acids Res.* **46**, D1144–D1149 (2018). 29099951 10.1093/nar/gkx1018 PMC5753333

[20] Ru, B., Tong, Y. & Zhang, J. MR4Cancer: a web server prioritizing master regulators for cancer. *Bioinformatics* **35**, 636–642 (2019). 30052770 10.1093/bioinformatics/bty658

[21] Ha, S. D. et al. Transcription-dependent targeting of Hda1C to hyperactive genes mediates H4-specific deacetylation in yeast. *Nat. Commun.* **10**, 4270 (2019). 31537788 10.1038/s41467-019-12077-w PMC6753149

[22] Langmead, B. & Salzberg, S. L. Fast gapped-read alignment with Bowtie 2. *Nat. Methods* **9**, 357–359 (2012). 22388286 10.1038/nmeth.1923 PMC3322381

[23] Zhang, Y. et al. Model-based Analysis of ChIP-Seq (MACS). *Genome Biol.* **9**, R137 (2008). 18798982 10.1186/gb-2008-9-9-r137 PMC2592715

[24] Magers, M. J., Udager, A. M. & Mehra, R. MiT family translocation-associated renal cell carcinoma: a contemporary update with emphasis on morphologic, immunophenotypic, and molecular mimics. *Arch. Pathol. Lab. Med.* **139**, 1224–1233 (2015). 26414466 10.5858/arpa.2015-0196-RA

[25] Cheng, J. et al. Computational analysis of pathological images enables a better diagnosis of TFE3 Xp11.2 translocation renal cell carcinoma. *Nat. Commun.* **11**, 1778 (2020). 32286325 10.1038/s41467-020-15671-5 PMC7156652

[26] Margolin, A. A. et al. ARACNE: an algorithm for the reconstruction of gene regulatory networks in a mammalian cellular context. *BMC Bioinforma.* **7**, S7 (2006). 10.1186/1471-2105-7-S1-S7 PMC1810318 16723010

[27] Sidhar, S. The t(X;1)(p11.2;q21.2) translocation in papillary renal cell carcinoma fuses a novel gene PRCC to the TFE3 transcription factor gene. *Hum. Mol. Genet.* **5**, 1333–1338 (1996). 8872474 10.1093/hmg/5.9.1333

[28] Martina, J. A. et al. The nutrient-responsive transcription factor TFE3 promotes autophagy, lysosomal biogenesis, and clearance of cellular debris. *Sci. Signal.* **7**, ra9 (2014). 24448649 10.1126/scisignal.2004754 PMC4696865

[29] Bakouny, Z. et al. Integrative clinical and molecular characterization of translocation renal cell carcinoma. *Cell Rep.* **38**, 110190 (2022). 34986355 10.1016/j.celrep.2021.110190 PMC9127595

[30] Sharabi, K. et al. Selective chemical inhibition of PGC-1α gluconeogenic activity ameliorates type 2 diabetes. *Cell* **169**, 148–160.e15 (2017). 28340340 10.1016/j.cell.2017.03.001 PMC5398763

[31] Choo, M. S. et al. Clinicopathologic characteristics and prognosis of Xp11.2 translocation renal cell carcinoma: multicenter, propensity score matching analysis. *Clin. Genitourin. Cancer* **15**, e819–e825 (2017). 28549862 10.1016/j.clgc.2017.04.015

[32] Argani, P. et al. Aberrant nuclear immunoreactivity for TFE3 in neoplasms with TFE3 gene fusions: a sensitive and specific immunohistochemical assay. *Am. J. Surg. Pathol.* **27**, 750–761 (2003). 12766578 10.1097/00000478-200306000-00005

[33] Lee, H. J. et al. Combination of immunohistochemistry, FISH and RT-PCR shows high incidence of Xp11 translocation RCC: comparison of three different diagnostic methods. *Oncotarget* **8**, 30756–30765 (2017). 28415646 10.18632/oncotarget.16481 PMC5458165

[34] Liberti, M. V. & Locasale, J. W. The Warburg effect: how does it benefit cancer cells? *Trends Biochem. Sci.* **41**, 211–218 (2016). 26778478 10.1016/j.tibs.2015.12.001 PMC4783224

[35] Škrtić, M. et al. Inhibition of mitochondrial translation as a therapeutic strategy for human acute myeloid leukemia. *Cancer Cell* **20**, 674–688 (2011). 22094260 10.1016/j.ccr.2011.10.015 PMC3221282

[36] Damayanti, N. P. et al. Therapeutic targeting of TFE3/IRS-1/PI3K/mTOR axis in translocation renal cell carcinoma. *Clin. Cancer Res.* **24**, 5977–5989 (2018). 30061365 10.1158/1078-0432.CCR-18-0269 PMC6279468

[37] Yin, X. et al. TFE3 fusions escape from controlling of mTOR signaling pathway and accumulate in the nucleus promoting genes expression in Xp11.2 translocation renal cell carcinomas. *J. Exp. Clin. Cancer Res.* **38**, 119 (2019). 30849994 10.1186/s13046-019-1101-7 PMC6408813

