# Supplementary material for: Comprehensive molecular characterization of TFE3-rearranged renal cell carcinoma
Source: Exp Mol Med. 2024 Aug 1;56(8):1807–15. doi: 10.1038/s12276-024-01291-2 (PMC11372160; doi:10.1038/s12276-024-01291-2)
Supplement: Supplementary file 1 — Supplementary materials [file 12276_2024_1291_MOESM1_ESM.pdf]

<Supplementary materials>

Comprehensive Molecular Characterization of TFE3-Rearranged Renal Cell Carcinoma

Supplementary Table 1. Primer sequences used in quantitative real-time PCR (qRT-PCR)

Gene	5'-Forward-3'	5'-Reverse-3'
TFE3	TGCCTGTGTCAGGGAATCTG	CGACGCTCAATTAGGTTGTGAT
PPARGC1A	TCTGAGTCTGTATGGAGTGACAT	CCAAGTCGTTCACATCTAGTTCA
E-Cadherin	TGAAGGTGACAGAGCCTCTGGAT	TGGGTGAATTCGGGCTTGTT
N-Cadherin	CCATCAAGCCTGTGGGAATC	GCAGATCGGACCGGATACTG
Vimentin	CCAAACTTTTCCTCCCTGAACC	GTGATGCTGAGAAGTTTCGTTGA
α-SMA	GTGTTGCCCCTGAAGAGCAT	GCTGGGACATTGAAAGTCTCA
GAPDH	GAAGGTGAAGGTCGGAGT	GAAGATGGTGATGGGATTTC

Supplementary Table 2. Top ten genes with high significances in RNA-seq and TFE3 ChIP-seq experiments.

Gene name	RNA-seq			ChIP-seq
	Log_2FC	P	P_{adj}	$-log_2P_{adj}$
GPR143	8.29	1.2×10^{-58}	1.8×10^{-54}	57.8
GSTP1	3.12	2.7×10^{-26}	6.1×10^{-23}	14.6
MBP	2.65	6.3×10^{-23}	1.1×10^{-19}	13.9
ABR	2.59	1.1×10^{-21}	1.6×10^{-18}	14.1
GCGR	7.93	2.8×10^{-20}	3.1×10^{-17}	20.4
SLC19A2	3.40	4.8×10^{-20}	4.8×10^{-17}	36.8
ASAH1	2.64	1.9×10^{-17}	1.2×10^{-14}	33.6
PFKB2	3.86	3.8×10^{-17}	2.4×10^{-14}	13.9
PPARGC1A	3.35	2.2×10^{-16}	1.2×10^{-13}	13.1
NDRG4	5.62	1.2×10^{-15}	5.7×10^{-13}	15.2

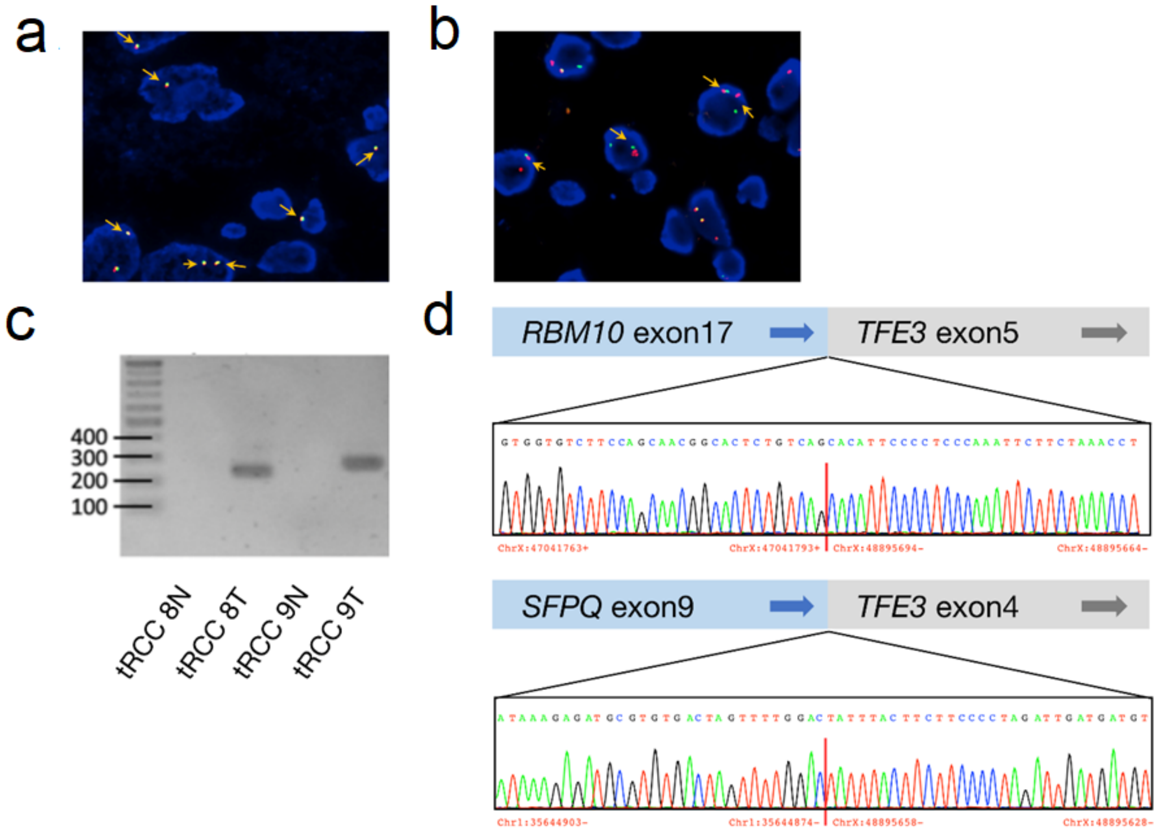

Supplementary Fig. 1. Identification of *TFE3* fusion in RCC patients. Representative fluorescence *in situ* hybridization (FISH) images of *TFE3* fusion negative (a) and *TFE3* fusion positive RCC (b). (c) Amplification of the cDNA fragment containing fusion point. (d) Sanger sequencing validation of the TFE3 fusion transcript from tRCC8 and tRCC9. Red vertical lines mark gene fusion points.

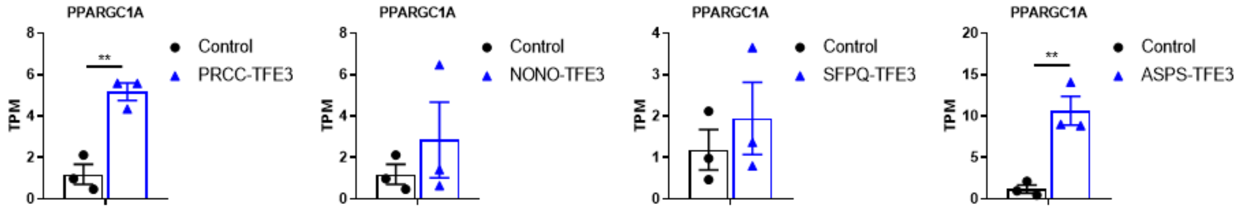

Supplementary Fig. 2. Increased expression of *PPARGC1A* in cells with *TFE3* fusion. Data were retrieved from Bakouny *et al., Cell Rep* 2022.

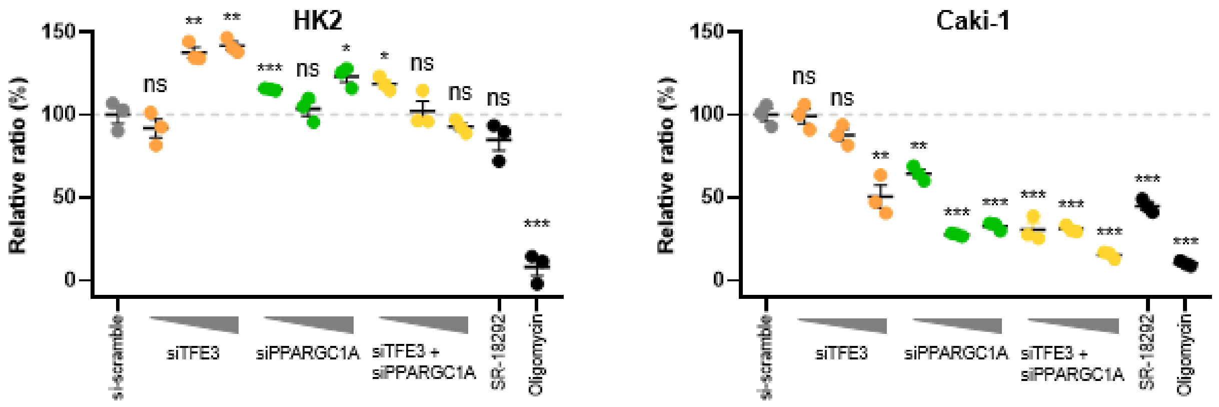

Supplementary Fig. 3. Effect of *TFE3* and *PPARGC1A* alterations on cell survival in normal kidney (HK2; left) and ccRCC (Caki-1; right) cells.

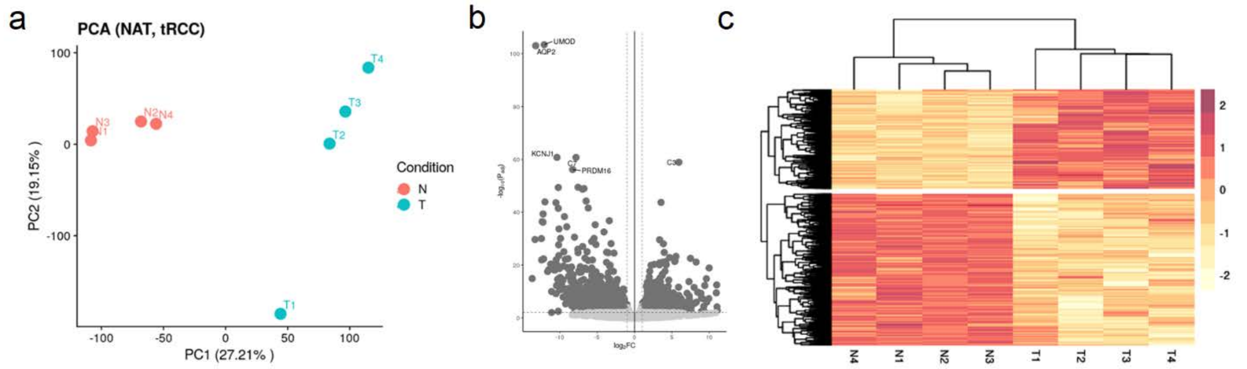

Supplementary Fig. 4. tRCC and normal transcriptomic profiles. (a) PCR plot of four paired tRCC and normal samples. (b) Volcano plot and (c) heatmap of 2,564 DEGs.

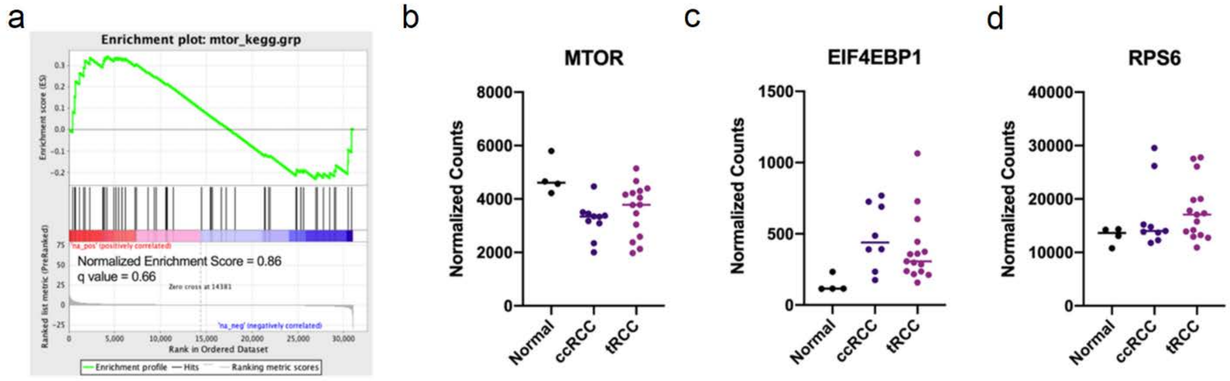

Supplementary Fig. 5 *MTOR* pathway in transcriptomic data of tRCC cells. (a) GSEA of *MTOR* pathway (KEGG) in tRCC tissues (b-d) Normalized RNA-seq counts of *MTOR* and its downstream targets *EIF4EBP1* and *RPS6*.
